# A Sensor Placement Approach Using Multi-Objective Hypergraph Particle Swarm Optimization to Improve Effectiveness of Structural Health Monitoring Systems

**DOI:** 10.3390/s24051423

**Published:** 2024-02-22

**Authors:** Muhammad Waqas, Latif Jan, Mohammad Haseeb Zafar, Syed Raheel Hassan, Rameez Asif

**Affiliations:** 1 Electrical Engineering Department, Iqra National University, Peshawar 25000, Pakistan; m.waqas@inu.edu.pk; 2 Computer Science Department, Iqra National University, Peshawar 25000, Pakistan; latifjan@inu.edu.pk; 3 Cardiff School of Technologies, Cardiff Metropolitan University, Cardiff CF5 2YB, UK; mhzafar@cardiffmet.ac.uk; 4 School of Computing Sciences, University of East Anglia, Norwich NR4 7TJ, UK; raheel.hassan@uea.ac.uk

**Keywords:** structural health monitoring, Multi-Objective Hypergraph Particle Swarm Optimization, Optimal Sensor Placement, Grey Relational Analysis, Fuzzy Decision Making

## Abstract

In this paper, a novel Multi-Objective Hypergraph Particle Swarm Optimization (MOHGPSO) algorithm for structural health monitoring (SHM) systems is considered. This algorithm autonomously identifies the most relevant sensor placements in a combined fitness function without artificial intervention. The approach utilizes six established Optimal Sensor Placement (OSP) methods to generate a Pareto front, which is systematically analyzed and archived through Grey Relational Analysis (GRA) and Fuzzy Decision Making (FDM). This comprehensive analysis demonstrates the proposed approach’s superior performance in determining sensor placements, showcasing its adaptability to structural changes, enhancement of durability, and effective management of the life cycle of structures. Overall, this paper makes a significant contribution to engineering by leveraging advancements in sensor and information technologies to ensure essential infrastructure safety through SHM systems.

## 1. Introduction

The introduction section provides an overview of structural health monitoring (SHM) and its importance in assessing and diagnosing infrastructure health. It highlights the advancements in sensor and information technologies that have revolutionized SHM. The section also introduces the novel Multi-Objective Hypergraph Particle Swarm Optimization (MOHGPSO) algorithm proposed in this paper.

The practice of developing and implementing strategies and procedures for the ongoing assessment and upkeep of an edifice’s structural integrity is termed SHM. The substantial costs associated with repairing and rehabilitating bridges and high-rise buildings underscore the importance of advancing structural reliability and integrity monitoring. Integrating SHM technologies can significantly prolong the lifespan of a structure, enhance security, and reduce restoration expenses. While the deterioration of system conduction is inevitable, it can feasibly be reversed, however, structural failure or loss of functionality can be prevented.

Non-destructive techniques (NDTs) are employed to detect local deterioration in Reinforced Cement Concrete (RCC) structures, such as the formation of fractures and corrosion. RCC is a type of concrete that contains reinforcement materials, such as steel bars, to enhance its strength and durability in structural applications.

SHM assesses the structure’s oscillations through damage detection schemes. Damaged structures exhibit different mass, stiffness, and damping values, affecting modal structure, strain energy, and inherent frequency [1]. The selection of appropriate sensor types and damage detection techniques is based on these features [2]: considering economic, environmental, and operational constraints [3]. SHM allows for the collection of intermittent or real-time continuous data, enabling the estimation of a building’s current health and future performance, and facilitating better preventative maintenance [4].

SHM is a multi-stage process reliant on prior stages for progression. Traditional wired systems use coaxial wires for data transport, ensuring data reliability and security on central servers. However, their cost-effectiveness is limited to smaller buildings or specific location studies. The overall cost of a wired network is determined by the size of the data collection system, leading to increased installation costs for large-scale structures [5]. To address this, there is a motivation to transition from wired to wireless structural monitoring.

**Figure 1:**
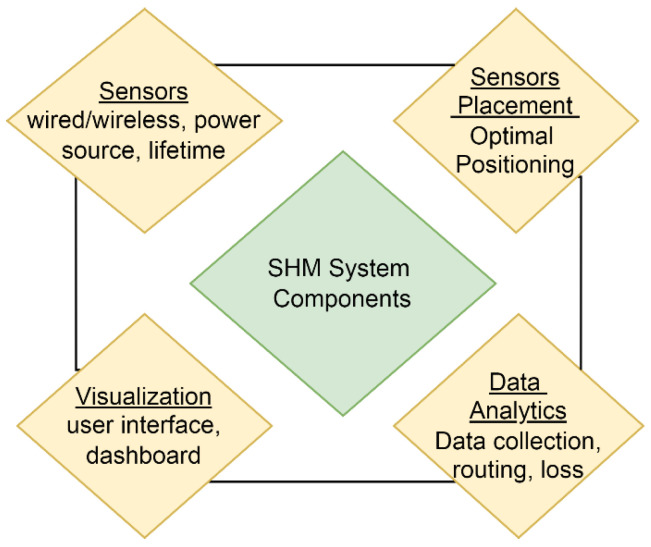
SHM’s major components.

The implementation of Wireless Sensor Nodes (WSNs) in structural health monitoring introduces various challenges, with each component in Figure 1 of SHM representing a research area. Effective sensor placement requires an in-depth understanding of the structure and the qualities gathered by the sensors, potentially involving optimization techniques, or drawing conclusions based on similar structures. Despite increased installation, maintenance, and weight costs, sensors on a structure can enhance the durability of an SHM system in case of crucial sensing node failures [6]. The efficiency of data collection and visualization relies on optimal sensor placement to conserve sensor nodes’ energy. Therefore, this article focuses on the positioning of sensor nodes as a key objective. The major contributions of this work, following the outlined motivation, are:

- A novel optimization algorithm with the concept of a hypergraph is developed for the optimal sensor’s placement in the structure.
- Multiple structural objectives are incorporated to decide the location preference, and a Pareto front with the non-dominated solutions in the archive is developed.
- A novel relational analysis is developed to determine the new solution’s entry in the archive of the Multi-Objective Hypergraph Particle Swarm Optimization algorithm.
- Fuzzy decision-making is used to obtain the single optimal solution from the archive.
- A spring–mass system and fixed wing of an airplane are used for the analysis.

The remainder of this paper consists of a study of the relevant literature in Section 2, followed by a discussion of the methodology being proposed in Section 3. In Section 4, we dive deeper into the multi-objective HGPSO and the innovative archive solution. Section 5 presents the analysis of the structural items, and Section 6 draws a conclusion based on this study. 

## 2. Literature Review

The literature review section discusses previous studies and research related to SHM systems. It mentions the use of automated sensor-based data-gathering strategies and storage module approaches for extracting sensor data and determining the extent of damage. It also highlights the development of OSP techniques for specific structures.

SHM is an advanced system that employs advanced sensing technology and automated data collection procedures to help predict the deterioration of a structure at an early stage. It is possible for businesses and researchers to apply this predictive study to gain a better understanding of the structure’s nature, standards, and bearing capacity under static and dynamic load conditions. The dynamic load of a structure can be calculated in addition to the static load resulting from its design or from the interactions between the structure and its environment. Dynamic loads can be calculated using a variety of methods, such as model identification using natural frequencies, time history analysis, or response spectra, among others. Olivera Lopez et al. [7] carried out a real-time examination of a 14-story structure in dynamically stressed conditions near the coastal region of Chile. In order to assess whether or not the structure could sustain a tsunami, Yanet et al. [8] identified that in order to more evenly distribute the weight and increase the sensor’s lifespan, the structure should be equipped with more sensors. The researchers conducted an analysis of the sensor’s lifespan. This strategy is referred to as the “communication technology load” in the industry.

Roghaei et al. [9] employed a static evaluation with SAP2000 software v17.0.0 and FEMA356 documentations while analyzing stress and deformation in a steel triple-story hospital construction. Zhou et al. [10] employed vibration analysis to detect damage, employing a more precise method known as the “hysteresis loop approach” to achieve their results (HLA). Using a 12-story reinforced concrete frame building to evaluate stiffness fluctuations, they discovered that the pinched technique was the most accurate because it properly predicted changes in cardinal frequencies close to 0.05 Hz. 

Pierdicca et al. [11] used an operational model analysis (OMA) approach in conjunction with a finite element model (FEM) for numerical simulation to analyze the dynamic behavior of a reinforced concrete school building; their findings were satisfactory in terms of both cost savings and accuracy, as demonstrated by their one-year monitoring.

Antunes et al. [12] found that the stiffness of an adobe masonry building decreased when the fundamental frequency dropped. The fundamental frequency of the test was found to have decreased by 48%. Sajedi et al. [13] conducted an experiment that employed 44 shaking tables. With the aid of OpenSees software (version 3.0.2), a three-story RC moment frame construction framework model with 180 ground motions and a 5400-time history analysis was produced. According to the simulation results, the incidence, location, and severity of damage were all predicted with 96, 87, and 90% accuracy. In the lab, damage classes could be predicted with a high accuracy of 92%. With the help of piezo materials, Gao et al. [14] conducted an experiment in which they recorded the time of arrival, carried out impedance analysis, and performed sweep frequency investigations through the demonstration of an embeddable tubular smart aggregate (TSA). For 2D concrete buildings, the outcomes were satisfactory.

Chatzis et al. [15] used an accelerometer to compute the intensity and determine the site of damage in a laboratory experiment involving shake tables. To determine the extent and location of damage, they used an improved T-SSID method using an unscented Kalman filter (UKF). The Bayesian time domain and the n4sid algorithm were used to compare T-SSID with UKF to determine which was better. Because of its quick approach to damage prediction, the UKF method was preferred above other damage prediction techniques.

Soltaninejad et al. [16] compared the short-time matrix pencil method (STMPM) with the discrete wavelet transform to produce a simulation for two neighboring structures to anticipate thumping under a unary-degree-of-freedom setup considering 36 cases. In fact, the data showed that STMPM was able to predict less severe damage rather than being affected by the size of the associated harm. The tool also helped in the prediction of sensor damage in both low- and high-resolution applications. The results of a one-month study conducted on the Sciri tower in Italy by Garca-Macas et al. [17] included the use of 12 accelerometers, and integrated ambient noise deconvolution interferometry, i.e., ANDI, along with multichannel optical analysis (OMA) to assess three frequency levels, estimated in the ranges of 200, 1000, and 5000 Hz. It was discovered that temperature fluctuations cause distortions, and the mode of wave propagation was investigated as a result.

Sun et al. [18] used three methods to determine the combined height of two buildings: system identification, wave propagation analysis using interferometry, and wave-based damage detection. They used the combined height of the two structures to present a model of a skyscraper, for example, the Al Harma Tower in Kuwait, which has 86 floors and a height of about 413 m. The dead weight of the building and the seismic response caused significant deformations in the structure. Morales-Valdez et al. [19] employed a microelectromechanical system (MEMS)-based accelerometer sensor (model code ADXL203E) to assess the disfigurement in a five-story building with dimensions of 60 × 50 × 180 cm by employing a wave propagation method to measure the force applied to the accelerometer sensor. According to the findings, the wave technique outperformed the modal analysis method in terms of stiffness reduction when only two factors were used: the minimal shear wave velocity and the Kelvin damping coefficient.

Valinejadshoubi et al. [20] focused on the purpose of extracting sensor data and determining the extent of the damage, and created the building information modeling (BIM). They used an automated sensor-based data-gathering strategy as well as a storage module approach in order to do this. Pachón et al. [21] developed a fine-tuned element model to forecast transient characteristics, such as mechanical vibrations and model classification, for an OSP technique for the Monastery of San Jeronimo de Buenavista in Seville, Spain.

The OSP functions mentioned in the sources are the methods or algorithms used to calculate and optimize the sensor placements in SHM systems. The MOHGPSO algorithm introduced in this paper utilizes six established OSP methods to generate a Pareto front of sensor placements, which is then analyzed using GRA and FDM techniques. The OSP functions aim to maximize the performance of the SHM system by considering factors such as modal strain energy, mode shapes, and the spatial relationships of model shapes. These functions play a crucial role in autonomously determining the relevant sensor placements without artificial interventions, ensuring the effectiveness and efficiency of the SHM system.

The energy matrix rank optimization techniques (SEMRO, KEMRO) and the constructive autonomy of target mode forms (EFIwm and EFI) serve as the foundation for four methodologies for analyzing the dynamic behavior of buildings: SEMRO, KEMRO, EFIwm, and EFI. KEMRO had a larger level of error in modal identification, but EFI had a lower level of error in the natural frequency. For the Italian Consoli Palace, Garca-Macas and Ubertini [22] used an automated anomaly detection system to foretell harm. Three models were taken into consideration: PCA, autoregression with an extrinsic input model (ARX), and multiple linear regression (MLR), in order to analyze local and global damage depending on the amplitude and resonant frequency.

To summarize, SHM utilizes advanced sensors and automated data collection to predict early-stage deterioration in structures. These systems aid in comprehending structure characteristics, standards, and load-bearing capacities under static and dynamic conditions. Dynamic loads are assessed through model identification, time history analysis, and response spectra. Real-time examination under dynamic stress allows the assessment of structures’ resilience to events like tsunamis. Increasing sensor deployment enhances weight distribution and extends sensor lifespan. Nonlinear static evaluation and vibration analysis detect stress, deformation, and damage. OMA and FEM analyze dynamic behavior, while piezomaterials and smart aggregates record arrival times and investigate frequency. Accelerometers and methods like the unscented Kalman filter compute intensity, locate damage, and predict it swiftly. Techniques such as the short-time matrix pencil method and discrete wavelet transform simulate impacts and predict damage to nearby structures. BIM and automated sensor-based data gathering extract sensor data for damage extent determination.

## 3. Methodology

The methodology section describes the proposed MOHGPSO algorithm and OSP. It explains the concept of a hypergraph and how it is incorporated into the algorithm. This section also mentions the use of GRA and FDM to analyze and archive the generated Pareto front.

The study presented in this paper demonstrates the utility of precursory ambient vibration test outcomes incorporated into material parameter uncertainties, providing a rigorous framework for comparing various OSP approaches for developing cost-effective, protracted monitoring systems. Among the FEM-based OSP methodologies examined, the EfI methods offer a solution that allows for the detection of fundamental frequencies with lesser inaccuracy while also ensuring a significantly low dispersion in the solutions. The sensor configurations obtained using the Driving Point Residue (DPR) method provide more information about the mode shapes, which aids in the prediction of a uniform sensor distribution throughout the edifice. The procedures in the average driving point residue (ADPR) and EfI-DPR methods augment this advantage on a larger scale. The Eigenvalue Vector Product (EVP) approach ensures the identification of invariance principles and undertakes a least square error analysis of data. The mode shape summation plot (MSSP) expediently identifies the highest deformation for the quickest assistance of the damage diagnostics. In our study, the required number of sensors is deduced, deploying the EfI-DPR method to find the optimal organization in each case. Then, EVP is deployed, which takes into account both a posited strain energy dispersion template and a surmised connectivity to form a well-established linear least squares problem involving the elemental stiffness matrix eigenvalues obtained. For the analysis of the reliance on the OSP solution, the results of the ambient vibration tests are employed.

### 3.1. Problem Statement

The limited supply of sensors and the conspicuous complexities of the typical issues in the contemplation project have extensive mutual incompatibility. Nearly all the proposed solutions are classifiable into two predominant approaches: one evinces single-objective optimization, while the other delineates a multi-objective viewpoint. However, single-objective methods typically do not reflect all performances of mode testing. This provokes the overlooking of certain potential optimal sensor placement methods in almost all such cases, and consequently, the respective methods are deemed ineffective in determining the aspired placement structure. Thus, it transpires as a prominent rationale behind the preference for multi-objective optimization in SHM over the single-objective approach. Just to augment this annotation, the subsequently generated respective series of solution sets, i.e., the Pareto front, require a trade-off of the distinct objectives, which is achieved to a greater degree by multi-objective optimization algorithms, as compared to single-objective techniques.

Notwithstanding the multi-objective optimization approaches, perforce presumes high auxiliary calculation costs. As a result, there are numerous proposals for improving the practical implications of multi-objective development techniques for OSP situations. The most promising suggestions propose transforming the multi-objective problem into a suitable single-objective conceptualization. Even the most basic mathematical operations, such as logarithm, product, exponent, or even summation, can realize various congregated OSP fitness methods with improved results. Nonetheless, such processes have a hidden proclivity to cause order discrepancies among the separate OSP methods. The focal strategies used to actualize the enhanced multi-objective optimal sensor placement techniques involve the diametrical transformation of the multiple objectives into a relevant single-objective format, which is either maneuvered according to determined weight factors (representation of the significance of each objective from the perspective of a decision maker) or is realized through the use of Pareto front optimization procedures.

The weight-factor-oriented strategy is simple and does not require algorithm adjustments because, following the aggregation step, a single-objective algorithm is used to identify the optimal solutions. In the absence of a decision-making paradigm, a Pareto optimum set can be formed by reiterating the single objective method with different weights. However, such an artificial setting may disturb the intrinsic characteristics of myriad methods in integrated optimization. Two noteworthy limitations become inevitable with this method. The normalization of each aim, first and foremost, demands assignment with a certain weight factor, or else the priority disparity and computational inaccuracies cannot be eliminated. Furthermore, in the absence of a supported computation or reference, the weight factor determinations are deemed arbitrary. In such cases, revising the weight factors for the combination of the objectives stipulates another function, requiring recalculation of the overall optimization and inflicting high computational costs. The pivotal drawback of this approach is that even symmetrically distributed sets of weight parameters may precipitate an asymmetrically dispersed collection of Pareto optimal solutions. Additionally, finding non-convex neighborhoods among the Pareto optimal front becomes unfeasible due to the summation of objectives using positive weights. 

On the contrary, an alternative bracket of multi-objective contingent escalation techniques utilizing interactive approaches makes it easier to incorporate decision-making preferences during optimization. The multi-objective formulation is preserved in this technique, but the programmed execution is interrupted to retrieve the decision-maker’s preferences. This strategy aids in avoiding the survey of undesired search space regions or the Pareto optimal front. Nonetheless, it requires human intervention and is therefore intrinsically protracted as compared to the aggregation or a posteriori algorithm. Convergence, i.e., the accuracy and speed of an approach in modeling Pareto optimal solutions, and coverage are two critical goals for ascertaining the Pareto optimal front employing a posteriori approach. As a result, extra weights or aggregation are rendered obsolete. As a result, the Pareto optimal solution set is determined in just one run, emphasizing the typical need for distributing solutions over the objectives to reinforce decision-making with multiple possibilities.

The ultimate goal of these propositions is to find a precisely explicit Pareto optimal solution set with as little participation in function evaluation as possible. This entails assigning Pareto optimum solutions to all objectives. As a result, a constructive algorithm is predicted to locate a symmetrically dispersed Pareto optimal front from a large number of different designs. However, the main constraint here is that coverage and convergence seem to be in contradiction, and so an approach is required to effectively counterbalance both in order to solve real-world multi-objective problems.

### 3.2. Proposed Methodology

SHM systems based on Operational Modal Analysis (OMA) and damage diagnostics are now authorized non-destructive techniques for assessing the real-time integrity of any architecture. OMA has the capacity to identify a building’s modal qualities. Modal update approaches aim to reduce disparities between practically inferred modal features and numerical model estimations, often based on the Finite Element Method (FEM), by fitting specific modal parameters.

Generally, SHM assemblies aim at controlling the structural performance of a building while recognizing any disfigurement and facilitating a condition-based conservation management system. To realize this particular objective, OSP methods provide prolific assistance by furnishing an efficient design of the optimized sensor orientation for an accurate determination of vibrational properties with fewer measurement points. The minimization of sensor count is a critical problem in this approach since it suggests protrusive diagnosis and has no impact on the building’s structural integrity. One of the crucial parameters of the design in diagnostics is the sensor placement for the efficient identification of the condition data.

Using data from sensors placed at the respective optimal configuration in the case of each of the OSP methods, the respective modal properties are determined, which are then treated as a vector of decision variables represented as:

(1) $$\bar{x}\;={\left[x_{1},\;x_{2},\;\ldots \;,\;x_{n}\right]}^{T}$$

Using this vector, the General Multi-Objective Optimization Problem (MOP) has been deduced in the form of the following vector function:

(2) $$\bar{f}\left(\bar{x}\right)\;=\;{\left[f_{1}\left(\bar{x}\right),\;f_{2}\left(\bar{x}\right),\;\ldots \;,\;f_{6}\left(\bar{x}\right)\right]}^{T}$$

Here, $f_{1}\left(\bar{x}\right)\;$represents the EfI function, $f_{2}\left(\bar{x}\right)$—the DPR, $f_{3}\left(\bar{x}\right)$—the ADPR, $f_{4}\left(\bar{x}\right)$—the EfI-DPR, $f_{5}\left(\bar{x}\right)$—EVP, and finally $f_{6}\left(\bar{x}\right)$ demonstrates the MSSP methods. The objective of this approach is to find the particular vector that can be represented as,

(3) $${\bar{x}}^{*}={\left[x_{1}^{*},\;x_{2}^{*},\;\ldots \;,x_{n}^{*}\right]}^{T}$$

which satisfies the following m inequality constraints:

(4) $$g_{i}\left(\bar{x}\right)\;\geq \;0\;\;\;\;\;\;\;i=1,\;2,\;\ldots \;,\;m$$

and also complies with the given p equality constraints:

(5) $$h_{i}\left(\bar{x}\right)\;=\;0\;\;\;\;\;\;\;i=1,\;2,\;\ldots \;,\;p$$

and thus, it can effectively optimize the respective deduced vector function $\bar{f}\left(\bar{x}\right)$, i.e., the current MOP in consideration.

In the context of evolutionary Multi-objective Optimization (EMO), the focal point lies in enhancing the efficiency of algorithms and data structures for storing non-dominated vectors. This involves sustaining diversity, reducing population size, and employing data structures for navigating unconstrained external archives of particles. The primary purpose of the external archive is to track non-dominated vectors or optimal position configurations discovered during the search process. Comprising an archive controller and a grid, the archive controller evaluates each vector in the primary population, incorporating only non-dominated ones based on Pareto dominance, while discarding dominated solutions. The proposed method introduces a novel archive controller utilizing GRA for this data comparison. When the external population exceeds the permissible capacity, an adaptive grid technique is invoked, creating space through hyper-cubes or hyper, depending on objective function range scaling. The objective parameter space in the archive is partitioned into these regions, uniformly distributed among the hyper-cubes/hyper-parallelepipeds. If the current insertion surpasses the grid’s constraints, a recalculation is performed, necessitating the relocation of individuals. The adaptive grid’s advantage lies in its significantly lower operational cost compared to niching, if not equivalent.

Thus, the accumulated position configurations are analyzed using the Pareto dominance tenets and incorporated into an enhanced HGPSO Algorithm that involves the amalgamation of Multi-Objective Optimization, i.e., MOHGPSO. This approach stands sui generis through variegation of the search proportions and also circumvents the precipitous convergence of particles. Here, the fitness eigenvalues of the obtained position orientations are calculated by a hypergraph. The concerning system has been presupposed to be a single cluster of all particles, where all particles are mutually interconnected. In a dynamic search space, each particle is treated as a node of the graph. Additionally, to aid in the realization of the aforementioned presumptions, a new term, namely, spectral cognition, has been introduced into the picture.

**Figure 2:**
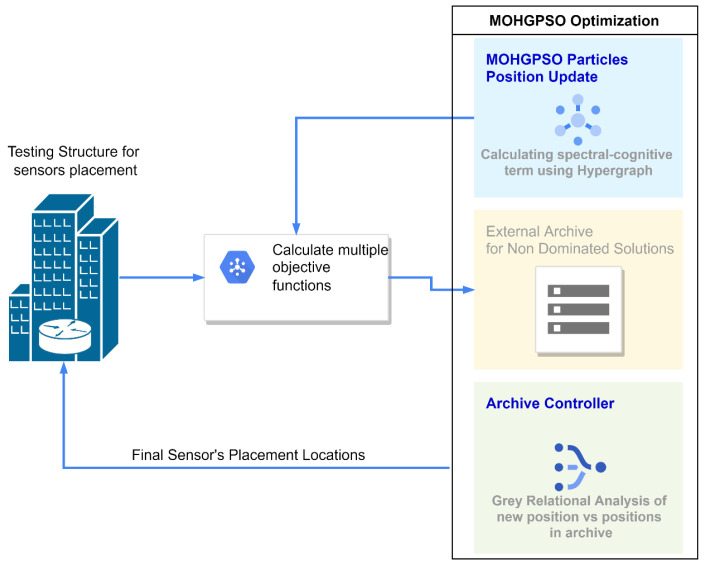
A schematic block diagram of the proposed methodology to optimally place the sensor nodes.

In Figure 2, the outline of the described methodology has been demonstrated in the form of a block diagram, which is further assisted by the following Algorithm 1.
**Algorithm 1**: Coarser pseudocode for the proposed methodologyInput: structure information from the FEM analysis, number of sensors m Output: optimal locations of the sensors
Get the random binary matrix for the sensor’s placement $X_{i\epsilon [0,1]}^{t},\;t=1,2\ldots maxiter$Calculate the multi-objective functions $F^{t}$ from the structural analysisStore the $X_{i\epsilon [0,1]}^{t}$ and $F^{t}$ in the external archive when t=1Update the particle’s position using the hypergraphed PSORepeat the step 2 and 3 for t=2Use the Grey relation analysis (GRA) on the archived particles to select the non-dominated solutionUpdate the archiveIf iterations are finished
a.Stop
Elseb.Repeat step 2 and 3EndSelect the single solution from the final archive using Fuzzy Decision modelling (FDM)


## 4. Proposed Solution

This section provides a detailed explanation of the proposed MOHGPSO algorithm and its application in determining the most relevant sensor placements. It discusses the incorporation of multiple structural objectives and the generation of a Pareto front with non-dominated solutions. The section also mentions the use of a novel relational analysis to decide new solutions’ entries in the archive.

### 4.1. Sensor Nodes’ Placement’s Objective Function

Generally, contrasting objectives are triggered when the rudimentary supply of sensors is faced with the incompatible complexity of routine multi-objective problems at hand. This provokes the generation of a series of solution sets, i.e., the Pareto front. These non-dominated fitness functions delineate a barter of the conflicting objectives, which is better obtained through multi-objective optimization algorithms, as compared to the single-objective ones [23].

Nevertheless, multi-objective optimization problems customarily demand high calculation costs [24]. Furthermore, choosing one or some of the optimal fitness functions from any of the optimum solution sets at or around the Pareto front is a diabolical task in and of itself. As a result, advancement has become a requirement for the practical utility of multi-objective optimization algorithms in OSP challenges.

The transition of multi-objective problems into single-objective problems is effective, but two restrictions are unavoidable [23]. To begin, each objective’s normalization must be assigned a weight factor, otherwise the priority discrepancy and computation errors cannot be eliminated. Second, if there is no supported analysis or reference, the weight factor decisions become arbitrary. In such circumstances, altering the weight factors for the combination of the objectives specifies another function, causing recalibration of the total optimization, and incurring large operational expenses. 

Nominating the analogous high-energy sensor orientations helps refine the ratio of signals regarding noise. On the other hand, subjugating the consequence of excessive or insufficient internal energy has been shown to be useful in process optimization. In the big picture, the traditional goals of optimizing sensor placements focus on three common perspectives: linear independence, energy, and average energy, and there are certain Optimal Sensor Placement approaches that show promise in achieving these. 

### 4.2. EfI Method

Effective Independence has been perceived as an iterative approach that ranks candidate sensor locations according to their contribution to the linear independence of the target modal partition. The EfI function strains upon the escalation of linear independence [25]. It is an adroit approach to $u\left(t\right)$, centered on the modal superposition theory, which is obtained via *N* mode shapes as:

(6) $$u\left(t\right)=\Phi q\left(t\right)+\omega =\sum_{i=1}^{N}\varphi_{i}q_{i}\left(t\right)$$

Here,

Φ: *n x n* matrix of modal shapes
$\varphi_{i}$: its *i-th* order
*n:* candidate sensor positions count
*N*: order number
$q\left(t\right)$: the generic modal coordinates
$q_{i}\left(t\right)$: its *i-th* order
ω: noise vector

Generally, the inverse operation of Equation (7) is used through the modal identification process [26] to recover the associated modal responses Φ from the system vibration signal q. When an efficient neutral interpolation is applied to E, the covariance of the erroneous outcome J is determined as follows:

(7) $$J=E\left[\left(q-\bar{q}\right){\left(q-\bar{q}\right)}^{T}\right]={\left[\frac{1}{\sigma^{2}}\Phi^{T}\Phi \right]}^{-1}=Q^{-1}$$

Here, Q is the Fisher Information Matrix (FIM), generally expressed as

(8) $$Q=\frac{1}{\sigma^{2}}\Phi^{T}\Phi =\frac{1}{\sigma^{2}}A_{0}$$

In order to achieve the optimum estimation, $A_{0}$ needs to be maximized. Furthermore, $E_{D}$ is calculated as:

(9) $$E_{D}=\Phi^{T}\Psi \lambda^{-1}{\left(\Phi \Psi \right)}^{-1}=\Phi {\left(\Phi^{T}\Phi \right)}^{-1}\Phi^{T}$$

Here, Ψ and λ are the analogous eigenvector and eigenvalue of $A_{0}$, respectively. Following a reiterative template, the minimal term in $E_{D}$ is rejected after each iteration while matching entities are deleted from the mode forms until the desired sensor count, *m*, is reached. The contributions of sensor sites to structural mode independence have been observed to be proportional to the value of $E_{D}$.

### 4.3. Driving Point Residue (DPR)

The DPR strategy [27] has promising prospects for determining a specific sensor position. It can be represented as an equivalence to modal participation factors, which assess the level of excitation or participation of each mode value in the overall response. The amplitudes of the resonance spikes in the frequency response function of a driving point are proportional to the magnitudes of the driving point residues. This is an energy-oriented OSP approach, and when DPR values increase, sensor placements become more constant. It has been established that:

(10) $$DPR=\Phi \;⊗\;\Phi \Omega^{-1}$$

Here, ⊗ is term-by-term matrix multiplication, and Ω denotes the circular frequency matrix. Each element of the *DPR* matrix represents the driving point residue contribution of that degree of freedom in a particular target mode.

### 4.4. Average Driving Point Residue (ADPR)

The Average Driving Point Residue [28] approach dispenses the measure of a point’s contribution to global performance. It is effective in reducing the effect of the zero-motion point. The ADPR in the *i*-th DOF for all N mode shapes can be determined using the equation:

(11) $$ADPR_{i}=\frac{1}{N}\sum_{j=1}^{N}DPR_{ij}$$

Here, $ADPR_{i}$ is the measure of participation of *i*-th *DOF*, and $DPR_{ij}$ determines the *i*-th DOF associated with the *j*-th mode order.

### 4.5. EfI-DPR Method

The EfI function achieves noteworthy efficiency in maximizing the lineal autonomy of the delegated modes. Notwithstanding, it does not take into account the energy of the whole assembly. Suboptimal sensor placements make mode detection harder in circumstances with a weak signal-to-noise ratio. The EfI-DPR [29] method has been demonstrated to overcome these shortfalls. The effective independence driving-point residue (EfI-DPR) method delivers an efficacious approach for optimal sensor placement applications, in which the EfI metrics are weighed, including the associated *DPR*, as shown below:

(12) $$E_{D\underline{\;\;}DPR}=E_{D}\;⊗\;DPR$$

Thus, this approach effectively balances both independence attributes and energy.

### 4.6. Eigenvalue Vector Product (EVP)

The eigenvalue vector product (EVP) [30] is a further energy-oriented OSP function, based on an empirically obtained flexibility matrix being projected out onto the strain energy redistribution in regional or local super elements. It takes into account both a posited link and a presumed strain energy distribution pattern while constructing a well-established linear least squares problem involving elemental stiffness matrix eigenvalues. Individual element or super element stiffness is proportional to these eigenvalues. This methodology incorporates the construction of modal degrees of freedom as derivatives of recorded sensor degrees of freedom to account for position offsets in practical sensor data. The following is the implementation:

(13) $$EVP_{i}=\prod_{j=1}^{N}\left|\Phi_{ij}\right|$$

Sensors are placed at locations with the largest EVP values to ensure the maximum vibration energy.

### 4.7. Mode Shape Summation Plot (MSSP)

The mode shape summation plot (MSSP) method for OSP can be perceived as an approach similar to the EVP. As demonstrated in [31], its implementation may involve the calculation of the sum of a set or subset of (target) modes, and for a given set of modes and within the selected modes (assembly or component), a user-specified number of modes with the highest (summed) deformation will be grouped. It is also an energy-based OSP method. Its customary utility is to select sensors in the initial case by deleting the low-energy positions, as follows:

(14) $$MSSP_{i}=\sum_{j=1}^{N}\left|\Phi_{ij}\right|$$

Here, *MSSP* values for the *i*-th *case* are calculated by considering the mode values $\Phi_{ij}$ in the target subset for each element from *j* = 1 to *N* specified by the user.

### 4.8. Multi-Objective OSP to Relational Objectives

The whole perception of GRA is pivoted on a specific concept of information. In this technique, situations with no information are supposed to be defined as black, while those with *perfect information* are deemed white. Nevertheless, both scenarios act as the idealized extrema, and the practical issues at hand are somewhat between them with *partial information* and are denominated as grey, hazy, or fuzzy. Thus, it can handle both quantitative and even qualitative data quite proficiently. This very attribute transpires as a legitimate advantage of GRA, making it a stark and more flexible and reliable strategy as compared to the other contemporary approaches that operate through heuristics or subjective judgements and can work with data given only in a certain format.

The grey relational analysis involves three concise steps for decision-making:

Step 1. Finding the grey relational grade.

A normalized matrix of fitness values is constructed to circumvent distortions caused by larger sample values of any element. The fitness value $f_{i}\left({\bar{x}}_{i}\right)$ of an individual particle, having six attributes $f_{i\in }\left({\bar{x}}_{i}\right)\left\{f_{i1}\left(\bar{x_{i}}\right),\;f_{i2}\left(\bar{x_{i}}\right),\;\ldots \;,\;f_{i6}\left(\bar{x_{i}}\right)\right\}$ as calculated by applying the objective function to its position vector, is then used in Algorithm 2 and is used to generate a comparable matrix for relational matrix generation as:

(15) $$Y_{ik}=\frac{f_{ik}-\min\left(f_{ik}\right)}{\max\left(f_{ik}\right)-\min\left(f_{ik}\right)}$$

Step 2. Figuring out the grey relational coefficient.

The grey relational coefficient determines the value of closeness between $Y_{ik}$ and $Y_{0k}$, i.e., the higher the value of the coefficient, the closer the two samples will be. It can be computed as:

(16) $$\gamma \left(Y_{0k},\;Y_{ik}\right)\;=\;\frac{\Delta_{min}\;+\;\zeta \Delta_{max}}{\Delta_{\mathrm{ik}}\;+\;\zeta \Delta_{max}}$$

Here, γis the grey relational coefficient between $Y_{0k}$ and $Y_{ik}$. ζ is the distinguishing coefficient, a random value between zero and one, which regulates the expansion and compression of relational coefficient. Using the values $\Delta_{ij}\;=\;\left|Y_{0k}\;-\;Y_{ik}\right|$, $\Delta_{min}$ and $\Delta_{max}$ are calculated as:

(17) $$\Delta_{min}\;=\;\min\left(\Delta_{ij}\right)$$

(18) $${\;\Delta }_{max}\;=\;\max\left(\Delta_{ij}\right)$$

Step 3. Employing the grey relational coefficient in decision making.

Using the equation grey relational coefficient from (16), the grey relational reward is calculated, which is used to select the higher relational samples. In our case, the betweenness degree $g\left(v\right)$ is calculated to generate a graph. The higher the value of *g(v)* for a sensor orientation, the higher its optimality. This concept is used to aid the tenets of the Pareto dominance implemented in the methodology and finally figure out the desired sensor orientation OSP. The process is depicted in the following Algorithm 2.
**Algorithm 2:** Optimality collation of sensor orientations using Grey Relational Analysis**Input**: Fitness value matrix f (1) Normalize the matrix f  using Equation (15) (2) For i = 1 : n (3)    a. For j = 1 : k $(4)\;\;\;\;\;\;\;i.\;\mathrm{Calculate}\;\mathrm{the}\;\mathrm{grey}\;\mathrm{relational}\;\mathrm{coefficient}\;\gamma_{ij}$ using Equation (16) (5)     b. end for (6) end for  (7) Generate a graph object from $\gamma_{ij}$ $(8)\;\mathrm{Calculate}\;\mathrm{the}\;\mathrm{between}\;g\left(v\right)$ for each element $(9)\;OSP_{indx}=\max g\left(v\right)$


### 4.9. Multi-Objective Hypergraph Particle Swarm Optimization (MOHGPSO) Algorithm

MOHGPSO is a novel algorithm introduced in this paper for OSP in SHM systems. It combines the concepts of MOO and PSO to address the challenges of sensor placement effectively. The algorithm employs six established OSP methods to generate a Pareto front, which represents a set of optimal solutions. The MOHGPSO algorithm utilizes a hypergraph to calculate the fitness eigenvalues of the obtained position orientations. This hypergraph represents a single cluster of interconnected particles, where each particle is treated as a node in the graph. The algorithm incorporates a dynamic search space where each particle’s fitness value is compared to others, and the difference is considered an edge between them in the hypergraph. The MOHGPSO algorithm autonomously determines the most relevant sensor placements in the combined fitness function without artificial interventions, showcasing its superior performance in optimizing sensor placements for SHM systems.

The following section of this paper includes a detailed overview of the proposed approach through a brief introduction to all the terminologies and concepts implemented. A brief synopsis of the PSO is followed by inception of the MOHGPSO algorithm. Thereafter, the decision-making methodology has been delineated, and finally, a summarizing algorithm has been provided to demonstrate the whole process.

PSO is among the stellar examples of bio-inspired evolutionary algorithms. Having very few hyperparameters makes it a candid approach in searching for an optimal solution in a given search space. The feature that distinguishes it from other optimization algorithms is that even the only objective function needed is independent of the gradient or any differential form of the objective. In spite of these noteworthy edges, PSO faces certain prominent shortcomings which entail modifications. Firstly, conventional PSOs are prone to the impulsive convergence of particles, i.e., far from the expected outcome of the objective function, thus compromising the efficiency and accuracy of the algorithm. Secondly, there is inadequate diversity, which affects the global search performance due to the large number of iterations required to find the globally optimal low value of cost function.

In recent years, hypergraphs have been widely used in some fields of computer science, such as image segmentation [32], data mining [33], and social network analysis [34]. A hypergraph is a generalization of an ordinary graph model where each hyperedge connects to an arbitrary number of hypervertices instead of only two. Thus, the hypergraph model facilitates designing group relations instead of only binary ones, i.e., it is applicable to problems with more than two variables or objects. The expediency of hypergraphs is associated with their higher connectivity as compared to their traditional counterparts.

A hypergraph-based particle swarm optimization has been validated to efficiently solve the problem of premature convergence in traditional PSOs and also to improve the diversity and global search performance of traditional PSOs through a reduction in the relevant number of iterations. This could be achieved through the introduction of a new direction vector to the position update in the exploration process of a vanilla PSO. The velocities of PSO in the case of a sequence optimization problem are defined as a series of swap operations based on a probabilistic update rule for the current position update.

Each particle has two attributes: velocity and location, represented as vectors $v_{i}$ and $x_{i}$, respectively. The fitness value $f_{i}\left(x_{i}\right)$ of a particle is calculated by applying the objective function to its position vector (location). The computed fitness values are then compared with their own previous locations or other particles’ locations, in order to obtain their respective individual personal best position ${\bar{p}}_{i}$ and the global best position $\bar{g}$. Then, the individual velocity and position of each particle are updated as follows:

(19) $$v_{i,t+1}\;=\;\omega \times v_{i}+c_{1}r_{1}\times \left({\bar{p}}_{i,t}-x_{i,t}\right)+c_{2}r_{2}\times \left({\bar{g}}_{i,t}-x_{i,t}\right)$$

(20) $$\mathrm{and},\;\;x_{i,t+1}\;=\;x_{i,t}+v_{i,t+1}$$

Here, ω, $c_{1}$ and $c_{2}$ represent constant weighting factors; the term ${\bar{p}}_{i,t}$ is the personal best location at time t; the term ${\bar{g}}_{i,t}$ represents the global best position of all particles, obtained through comparison of fitness values of particles with each other. The terms $r_{1}$ and $r_{2}$ are two independent random variables in the range [0, 1].

Equation (19) represents the exploration step of the PSO and is modified in the hypergraph PSO (HGPSO) approach. A new fourth parameter is added to the equation, as shown in Equation (21):

(21) $$v_{i,t+1}\;=\;\omega \times v_{i}+c_{1}r_{1}\times \left({\bar{p}}_{i,t}-x_{i,t}\right)+c_{2}r_{2}\times \left({\bar{g}}_{i,t}-x_{i,t}\right)+c_{3}r_{3}\times \left({\bar{h}}_{i,t}-x_{i,t}\right)$$

**Figure 3:**
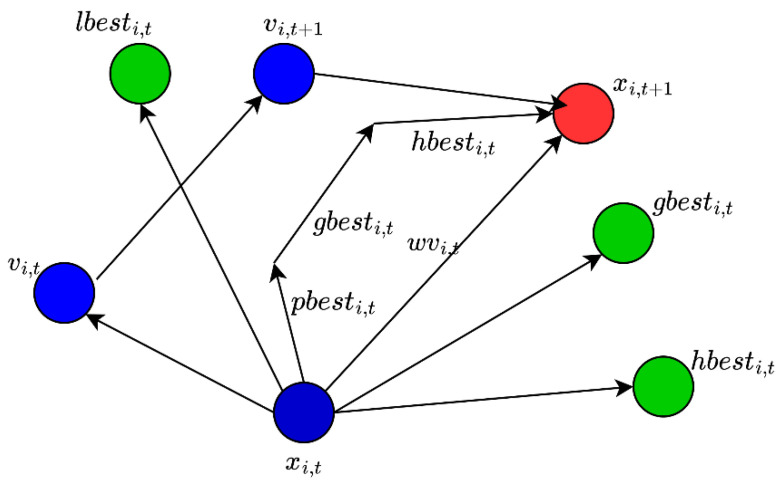
Particles’ positions update in Hypergraphed Particle Swarm optimization.

Here, ${\bar{h}}_{i,t}$ is the centroid of the particles in the hypergraph generated by the current fitness values of the particles. The update process of HGPSO is shown in Figure 3.

Here, the concept of hypergraph generation is inspired by TTM clustering in hypergraph theory. An adjacency matrix of m×m is used to obtain the weights of each particle’s connection to another. The particle’s fitness in any iteration is a vector quantity, and it has to be converted into an adjacency matrix using the nearest neighbor calculation scheme.

(22) $$A_{m\times m}=Adjacency\left(f_{i}\left(x_{1}\right),\;\ldots \;,\;f_{i}\left(x_{m}\right)\right)$$

where, A is the adjacency matrix of the vector of costs of the particles in the swarm, then a hypergraph k is calculated for A to obtain an eigenvector using equation (23) as:

(23) $$Par_{i\in \left\{1,\;\ldots \;,\;k\right\}}=\frac{1}{z!}Trace\left(A\;\times_{1}\;Y^{{\left(1\right)}^{r}}\;\times_{2}\;Y^{{\left(2\right)}^{r}}\;\ldots \;\times_{z}\;Y^{{\left(z\right)}^{r}}\right)$$

Here, $\times_{l}$ is the model-l product and $\;Y^{{\left(1\right)}^{r}}$, $\;Y^{{\left(2\right)}^{r}}\;Y^{{\left(2\right)}^{r}},\;\ldots \;,\;Y^{{\left(z\right)}^{r}}\;\in \;R^{k\times z}$ which represents the number of CMs connected to each node $n_{i}$ for each vertex $Y^{i\in \left\{1,\;2,\;\ldots \;,\;z\right\}}$ as:

(24) $$Y^{i}=\frac{1}{\sum_{{\;}_{k}^{\;}}CM\left(n_{i}\right)}$$

The centroid of k is calculated using k-means clustering.

In the proposed methodology, the congregated position orientations are maneuvered using the Pareto dominance tenets and incorporated into an enhanced Hypergraph Particle Swarm Optimization Algorithm which involves the coalescence of Multi-Objective Optimization, i.e., MOHGPSO. Certain terminologies, which have been implemented later in the study, are described briefly:

(i) Global Minimum: For a given function $f\;:\;\Omega \;\subseteq \;R^{n}\;\to \;R,\;\Omega \;\neq \;\emptyset ,$ if $\bar{x}\;\in \;\Omega$, and, more importantly, $\forall \bar{x}\;\in \;\Omega \;:\;f\left({\bar{x}}^{*}\right)\;\leq \;f\left(\bar{x}\right)$, the global minimum is estimated to be given by

(25) $$f^{*}\;≜\;f\left({\bar{x}}^{*}\right)\;>\;-\infty$$

Here, ${\bar{x}}^{*}$ represents the global minimum solution, f—the objective function, and Ω is the set representing the feasible region $\left(\Omega \;\in \;S\right),$ where, S includes the entire search space.

(ii) Pareto Dominance: If two vectors, one represented by $\bar{u}\;=\;\left(u_{1},\;u_{2},\;\ldots \;,\;u_{k}\right)$ and the other by $\bar{v}\;=\;\left(v_{1},\;v_{2},\;\ldots \;,\;v_{k}\right)$, respectively, are mutually related such that the objective values of $\bar{u}$ are no worse than those of $\bar{v}$, and are strictly better than the latter for at least one of the obtained solution elements, for any given objective, then vector $\bar{u}$ is said to dominate vector $\bar{v}$. In a nutshell: $\bar{u}\;≼\;\bar{v}$, i.e.,

(26) $$\forall i\;\in \;\left\{1,\;2,\;\ldots \;,\;k\right\},\;u_{i}\;\leq \;v_{i}\;\;\;\;\;\;\;\wedge \;\;\;\;\;\;\;\exists i\;\in \;\left\{1,\;2,\;\ldots \;,\;k\right\},\;u_{i}\;<\;v_{i}$$

(iii) General Multi-Objective Optimization Problem (MOP) and Pareto Optimal Set: The objective of this approach is to find a vector represented by,

(27) $${\bar{x}}^{*}={\left[x_{1}^{*},\;x_{2}^{*},\;\ldots \;,x_{n}^{*}\right]}^{T}$$

such that, it satisfies the following m inequality constraints

(28) $$g_{i}\left(\bar{x}\right)\;\geq \;0\;\;\;\;\;\;\;i=1,\;2,\;\ldots \;,\;m$$

and also complies with the given p equality constraints

(29) $$h_{i}\left(\bar{x}\right)\;=\;0\;\;\;\;\;\;\;i=1,\;2,\;\ldots \;,\;p$$

and thus, effectively optimizes the vector function

(30) $$\bar{f}\left(\bar{x}\right)\;=\;{\left[f_{1}\left(\bar{x}\right),\;f_{2}\left(\bar{x}\right),\;\ldots \;,\;f_{k}\left(\bar{x}\right)\right]}^{T}$$

Here, $\bar{x}$ represents the vector of decision variables and is computed as

(31) $$\bar{x}\;={\left[x_{1},\;x_{2},\;\ldots \;,\;x_{n}\right]}^{T}$$

For a given MOP $\bar{f}\left(x\right)$, the Pareto optimal set $\left(P^{*}\right)$ is estimated as:

(32) $$P^{*}\;:=\;\left\{x\;\in \;\Omega \;\;:\;\neg \exists x^{\prime }\;\in \;\Omega \;\bar{f}\left(x^{\prime }\right)\;≼\;\bar{f}\left(x\right)\right\}$$

(iv) Pareto Front:

A Pareto front represents a set of optimal solutions that cannot be improved in one objective without sacrificing performance in another objective. In the context of sensor placement for SHM systems, achieving a Pareto front is significant because it allows decision-makers to evaluate and select sensor placements based on multiple criteria simultaneously. By generating a Pareto front using the MOHGPSO algorithm, the proposed approach in this paper enables the identification of sensor placements that offer a balance between different objectives, such as maximizing data collection efficiency, conserving sensor nodes’ energy, and enhancing the durability of the SHM system. The comprehensive analysis of the Pareto front using GRA and FDM provides insights into the relative performance of different sensor placements, allowing decision-makers to make informed choices without artificial interventions. Ultimately, achieving a Pareto front in sensor placement optimization helps maximize the effectiveness and efficiency of SHM systems, ensuring the safety and longevity of critical infrastructure.

Usually, it is non-viable to realize an inquisitive linear or superficial expression containing nondominated values. The routine process of inducing the Pareto front is through the computation of the workable points Ω with the analogous $f\left(\Omega \right)$. With a substantial count, the determination of the aspired points becomes feasible. For a given MOP $f\left(\bar{x}\right)$ and Pareto optimal set $P^{*}$, the Pareto front $\left(PF^{*}\right)$ is defined as:

(33) $$PF^{*}\;:=\;\left\{u\;=\;f\;=\;\left(f_{1}\left(x\right),\;\ldots \;,\;f_{k}\left(x\right)\right)\;:\;x\;\in \;P^{*}\right\}$$

(v) Pareto Optimality: Conventionally, it is evaluated apropos the whole decision variable space (unless otherwise specified). For a point represented by ${\bar{x}}^{*}\;\in \;\Omega$ to be Pareto optimal, it is imperative that there exists no realizable vector that can decrease some criterion without causing a simultaneous increase in at least one other criterion. Thus, for every ${\bar{x}}^{\;}\in \;\Omega$ and $I\;=\;\left\{1,\;2,\;\ldots \;,\;k\right\}$

(34) $$\mathrm{that}\;\mathrm{either}\;\;\;\forall_{i\in I}\left(f_{i}\left(\bar{x}\right)\;=\;f_{i}\left({\bar{x}}^{*}\right)\right)$$

(35) $$\mathrm{or}\;\exists i\;\in \;I\mathrm{such}\;\mathrm{that},\;\;f_{i}\left(\bar{x}\right)\;>\;f_{i}\left({\bar{x}}^{*}\right)$$

Pareto optimal solutions are often also referred to as noninferior, admissible, or efficient solutions, while their analogous vectors are called nondominated.

The optimal placement of WSN depends on several factors. These objectives can be achieved by optimal placement, either by weighing all problems or by Pareto’s optimal solution as a multi-objective problem. The MOHGPSO is a novel proposed optimization algorithm that diversifies the solution search and avoids the premature convergence of particles. To continue with the MOHGPSO, the velocity update of the conventional PSO has to be studied first. The velocity in a conventional PSO is updated as:

(36) $$V_{j}^{i}=V_{j}^{i-1}+c_{1}\times rand\times (pbest_{j}^{i-1\;}-prvPos_{j}^{i-1})+c_{3}\times rand\times (gbest_{j}^{i-1\;}-prvPos_{j}^{i-1})$$

where, i and j represent the current iteration and *j-th* particle. The pbest and gbest are the local and global positions of the particles in the PSO. The first term in the above Equation (36) adds momentum to the particle. The second term is the cognitive term, which motivates the particle to move towards the local best position, and the last term is the collective term, which enhances the search capability nearer to the global best particle’s position.

**Figure 4:**
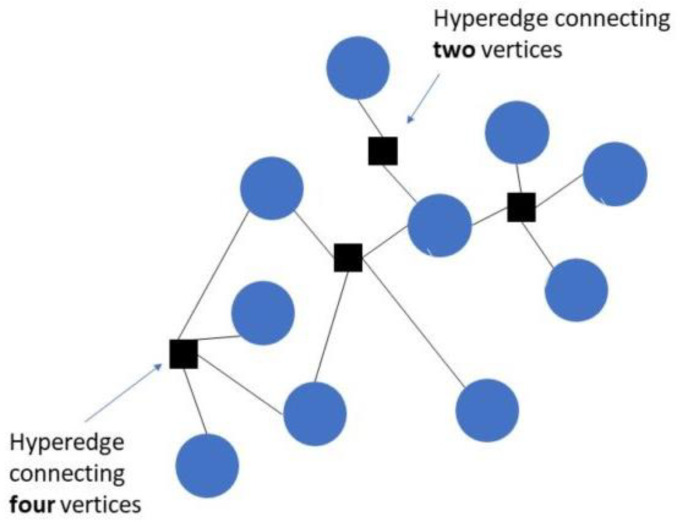
Hypergraph representation of PSO particles.

To improve the convergence and avoid the local minima problem of MOPSO, we hereby introduce Hypergraph PSO. In HGPSO, the eigenvalues of the fitness values of all particles in an iteration are calculated by a hypergraph. Considering a single cluster of all particles, every particle is connected to another. Considering the arrangement of PSO’s in the dynamic search space, each particle is considered a node of the graph. The difference in fitness value between two particles is considered the edge between them, as shown in Figure 4.

Hypergraphs are greatly pivoted on the dynamic evolution process, substantiating the exploration of the dynamic analysis of complicated networks. The further advantage of the hypergraph theory involves ensuring point and edge uniformity. Finally, they aid in clearly expressing the relationship between the nodes and edges.

In MOHGPSO, the external repository is maintained at the end of every iteration, which houses the best particles so far. It comprises the archive controller and the grid. The archive controller examines each vector, found in an iteration in the primary population of the algorithm, and compares it to the existing contents of the repository individually on the basis of Pareto dominance—to append only the nondominated ones. Conversely, if the archive holds solutions that are dominated by the new element they are canonically discarded. The unique archive controller in the proposed method deploys Grey Relational Analysis (GRA) for this collection of data. Eventually, when the external population surmounts the allowable capacity, the implementation of the adaptive grid procedure is initiated, which in fact is a space formed by hyper-cubes or hyper-parallelepipes, depending on whether the ranges of the objective functions are scaled or not, respectively. In the repository, objective parameter space is partitioned into these regions, which are dispersed uniformly among the greatest number of hyper-cubes or hyper-parallelepipes possible. The repository size is defined by the hit-and-trial scheme. We consider here that 10 particles can be housed in the repository.

This repository’s best fitness value for each objective (since it is a multi-objective problem) is extracted and subtracted from each population’s fitness value. The adjacency matrix is thus created, and hyperspectral clustering is performed to obtain the centroid of the population. This centroid keeps the position of each particle nearer to the best values in the repository. So, we add a fourth term in Equation (1) as:

(37) $$V_{j}^{i}=V_{j}^{i-1}+c_{1}\times rand\times \left(pbest_{j}^{i-1\;}-prvPos_{j}^{i-1}\right)+c_{3}\times rand\times \left(gbest_{j}^{i-1\;}-prvPos_{j}^{i-1}\right)+c_{4}\times rand\times (gCentroid_{j}^{i-1}-prvPos_{j}^{i-1})$$

This fourth term is called the spectral-cognition term.

The steps to calculate the spectral-cognitive term in MOHGPSO are as follows:

(a) The particles in the repository that are best so far and the current position of all particles and their corresponding fitness values are used.
(b) Calculate the best fitness value for each objective in the multi-objective from the repository.
(c) Subtract that best value from each particle’s fitness value.
(d) Generate the adjacency matrix by following the nearest neighbor approach
(e) Use the hypergraph calculation of eigenvalues.
(f) Find the centroid position among all particles by the k-means clustering of eigenvalues calculated in step 5.

Subtract the centroid position from each of the particle’s current positions with a weighted value as in Equation (37).

In the proposed method, GRA is preferred over sorting based on domination level. One of the pivotal causes behind this is that the latter approach, although it promotes extrapolation, compromises with the quality of the grade of the non-dominated solutions. However, GRA ensures a noteworthy reduction in the probability of damaging the non-dominated solutions during optimization. The best non-dominated solutions are maintained even while abruptly updating the variables using several operators. This approach effectively supports superior exploration and convergence.

In the conclusive steps of MOHGPSO optimization, a set of optimal solutions will be accumulated in the external archive. Each component class has a number of properties that are deemed important in the decision-making process. Furthermore, there are some critical properties that are not well understood yet which are essential for the component to operate. Thus, fuzzy decision-making is used to direct the decision-making workflow towards finding the desired answer without imposing any required precondition of extensive understanding of the component type (FDM).

The process involves establishing an understanding of the needs in the respective decision-making, in our case, the optimality of the solutions. Then, the construction of the membership functions. Each functional optimization metric is interpolated in the range of 0–1 during the FDM process. As in Equations (38) and (39), the best orientation index is determined by the min–max of the normalized values.

(38) $${\bar{F}}_{k}=\left\{\begin{array}{c} 1,\;\;\forall \;F_{k}\leq F_{k}^{min}\;\\ \frac{{(F}_{k}^{max}-F_{k})}{\left(F_{k}^{max}-F_{k}^{min}\right)}\;\forall \;F_{k}^{min}<F_{k}>F_{k}^{max}\;\\ 0,\;\;\forall \;F_{k}\geq \;F_{k}^{max} \end{array}\right.$$

(39) $$OSP_{index}=\max\left(\min\left({\bar{F}}_{1},\;{\bar{F}}_{2},\;{\bar{F}}_{3},\;{\bar{F}}_{4},\;{\bar{F}}_{5},\;{\bar{F}}_{6}\right)\right)$$

where, ${\bar{F}}_{k}$ linear fuzzy membership for $k^{th}$ optimality metric where, $k\in \left\{1,\;2,\;3,\;4,\;5,\;6\right\}$, $F_{k}^{min}$, and $F_{k}^{max}$ are the minimum and maximum of $k^{th}$ optimality metric. The best optimal solution index is computed as in Equation (37), and that orientation is returned from the archive to present as the expected outcome.

Following this, GRA was used to obtain the improved non-dominated set of this relatively recently established multi-objective model. The key to the desired answer, however, has been agreement among the analyzed metrics. It could be used to highlight how far a solution is from the group’s preferred solution. To compensate for uncertainty in preference, robustness is proposed as a gauge of the capacity to cope with change in preference. As a result, the suggested approach takes both consensus and robustness into account.

### 4.10. Combination of HGPSO with GRA and FDM for Generating a Pareto Front

The MOHGPSO algorithm combines classical algorithms, such as the Hypergraph Particle Swarm Optimization (HGPSO), with Grey Relational Analysis (GRA) and Fuzzy Decision Making (FDM) to generate a Pareto front for optimal sensor placement. HGPSO, as a component algorithm, contributes to the overall performance of the MOHGPSO algorithm by leveraging the concepts of particle swarm optimization to explore the search space and find optimal solutions. GRA is used to systematically analyze and archive the Pareto front obtained from the OSP methods, providing insights into the relative performance of different sensor placements. FDM is employed to make fuzzy decisions based on the analyzed Pareto front, allowing decision-makers to determine the most relevant sensor placements in the combined fitness function without artificial interventions. The combination of HGPSO, GRA, and FDM enhances the algorithm’s ability to autonomously determine optimal sensor placements and generate a Pareto front, showcasing its superior performance in optimizing sensor placement for Structural Health Monitoring systems.

The proposed method has been summarized into certain fundamental steps in the form of Algorithm 3.
**Algorithm 3:** SHM Analysis using MOHGPSO, deploying GRA and FDMInput: structure information from the FEM analysis, number of sensors m.
Output: optimal locations of the sensors.(1)Get the random binary matrix for the sensor’s placement $X_{i\epsilon [0,1]}^{t},\;t=1,2\ldots maxiter$(2)$\mathrm{Calculate}\;\mathrm{the}\;\mathrm{multi}-\mathrm{objective}\;\mathrm{functions}\;F^{t}$ form the structural analysis(3)$\mathrm{Store}\;\mathrm{the}\;X_{i\epsilon [0,1]}^{t}$ and $F^{t}$ in the external archive when t=1(4)Update the particle’s position using HGPSO
Input: epoch size, swarm size m*,* ω*,* a*,* $a_{2}$*,* $a_{3}$Initialize: initial position of the swarm
(a)Calculate q, k*,* s*,* $f(x_{i})$ for the initial positions(b)do
(i)for each sequence x in the swarm do (ii)Update the velocity using $v_{i,t}=V_{i,t+1}(x,\;q,\;k,\;s,\;\omega ,\;a_{1},\;a_{2},\;a_{3})$(iii)Calculate new position ${\bar{x}}_{i,t}$ to update particle’s position(iv)If $f(\bar{x})$ Is better than f(x) then
(1)$f\left(x\right)=f(\bar{x})$(2)$x=\bar{x}$
(v)end_if(vi)If $f(\bar{x})$ Is better than f(k) then
(1)$f\left(k\right)=f(\bar{x})$
(vii)End_for(viii)Calculate $\bar{s}$ for current epoch and particle positions (ix)If ($\bar{s}$ Is better than s)
(1)$s=\bar{s}$
(x)end_if(xi)while (number of epochs are not satisfied) 

(5)Repeat the steps 2 and 3 for t=2(6)Use the Grey relation analysis (GRA) on the archived particles to select the non-dominated solution
(a)***Input***: Calculated Fitness value matrix f$\;\mathrm{using}\;F^{t}$(b)Normalize the matrix fusing Equation (15)(c)For i = 1 : n
(i)For j = 1 : k(1)Calculate the grey relational coefficient $\gamma_{ij}$ using Equation (16)(ii)end for
(d)end for(e)Generate a graph object from $\gamma_{ij}$(f)$\mathrm{Calculate}\;\mathrm{the}\;\mathrm{betweenness}\;g\left(v\right)$ for each element(g)$OSP_{indx}=\max g\left(v\right)$
(7)Update the archive with the obtained value, if needed(8)If iterations are finished
(a)Stop(9)Else
(a)Repeat steps 2 and 3(10)End(11)Select the single solution from the final archive using Fuzzy Decision modelling (FDM) as described through Equations (38) and (39)

## 5. Results and Discussions

The results and discussions section presents the analysis of structural items using the proposed algorithm. It discusses the outcomes obtained through different OSP methods and highlights the superior performance of the proposed approach. This section also includes figures and tables to support the findings.

### 5.1. Evaluation Parameters

After obtaining the conclusive results of the sensor orientations through the different OSP methods, five sensor placement benchmarks were to be verified to check the all-round performances vis-à-vis the sensor distribution, orthogonality, linear independence, energy, and redundant configuration.

#### 5.1.1. Determinant (DET) of FIM

The determinant is used as an evaluation approach for the coupling of mode shapes in OSP methods. The performance of a particular OSP method is directly proportional to the value of the determinant, which is calculated based on the spatial relationship of model shapes. The higher the determinant value, the better the performance of the OSP method in terms of coupling mode shapes and resistance against noise. The determinant is a measure of structural stiffness and plays a crucial role in assessing the performance and effectiveness of sensor placements in SHM systems. In a limited set of coordinates, the FIM determinant represents the trustworthiness of the data. By maximising the determinant, the EfI technique chooses the OSP configuration. Because it has the same relevance as a reiterative format, it is also effective to use a determinant to test the accuracy of the sensor locations as follows:

(40) DET=det⁡(Q)

The performance of the particular OSP is directly proportional to the value of the DET.

#### 5.1.2. Mean Value of Off-Diagonal Entries of MAC

It is an effective evaluation approach for the coupling of mode shapes utilizing the spatial relationship of model shapes, which is expressed as follows:

(41) $${MAC}_{ij}=\frac{{\left(\varphi_{i}^{T}\varphi_{j}\right)}^{2}}{\left(\varphi_{i}^{T}\varphi_{i})(\varphi_{j}^{T}\varphi_{j}\right)}$$

where, $\varphi_{i}$ and $\varphi_{j}$ are the *i*-th and *j*-th column in Φ.

Here, ${MAC}_{ij}$ demonstrates the cosine of the angle formed by the measured modes’ two vectors. Because a bigger space angle implies more obvious shape vectors, optimal sensor sites are acquired by lowering the proportions of the maximal off-diagonal MAC, generally derived by averaging the off-diagonal aspects.

(42) $$MAC=\frac{1}{n(n-1)}\sum_{\begin{array}{c} i=1\\ i\neq j \end{array}}^{N}\sum_{j=1}^{N}{MAC}_{ij}$$

Optimality of the configuration is inversely proportional to the MAC measure.

#### 5.1.3. Modal Strain Energy (MSE)

The modal strain energy (MSE), i.e., energy associated with sensor arrangement, is used to augment the weak signal-to-noise ratio in the OSP scenario, which is described as follows:

(43) $$MSE=\frac{1}{2}\sum_{i=1}^{N}\varphi_{i}^{T}K\varphi_{i}$$

where, K is the matrix accounting for the structural stiffness. The higher the MSE measure, the higher the resistance against noise.

#### 5.1.4. SDI

Notwithstanding its widespread use, the EfI approach has several significant flaws, including cluster orientations that repeatedly arise when the sensor count surpasses that of the recorded modal shapes, which can lead to spatial correlation and significant resource waste. As a result, the following SDI is shown to indicate the dispersed sensor placement:

(44) $$SDI=\frac{\mu \sum_{i=1}^{m}\min{}_{D_{ij}}}{2A}$$

where, μ is the mean distance between all the sensors and their center, defining the dispersion, $min(D_{ij})$ represents the separation of each sensor from its nearest neighbor, and A represents the structural area diagnosed, and dispersion in the distribution is directly proportional to the value of SDI, i.e., the higher the value, the lesser the redundancy information. Nevertheless, the indices particularly deliver the geometry dispersion data, overlooking any dynamic contributions.

The ratio of similar positions (RSP), achieved through comparison of various OSP functions.

The lack of an evaluation index for the eventual sensor orientation outcomes achieved through the various OSP approaches necessitates the establishment of this last criterion for RSP comparison.

(45) $$RSP=\frac{\sum_{i=1}^{k}l_{i}}{mk}$$

where, $l_{i}$ is the number of identical sites attained by one method and another. The more general the sensor position sets in the similar OSP approaches, the higher the value of this index.

#### 5.1.5. Analysis

During the course of simulation, a two systems spring–mass system and the fixed wing of an aeroplane are presented for optimal sensor placement by the proposed multi-objective methodology.

### 5.2. Spring–Mass System

**Figure 5:**
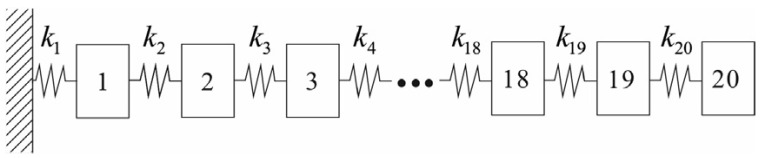
A spring–mass setup demonstrating 20 degrees of freedom.

**Figure 6:**
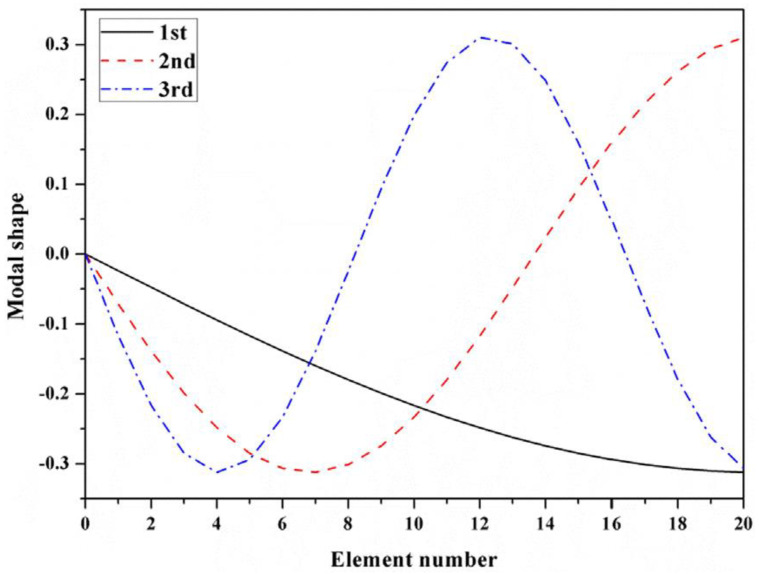
Depicted modal shapes of the spring-mass system.

The proposed set-up can be perceived to be analogous to a spring–mass system with, let us say, n = 20 DOFs. As for the case, the parameters are considered to be $k_{i}\;=\;1000\;N/m$ and $m_{i}=1\;kg,\;\left(i=1,2,3,\;\ldots \;,\;20\right)$. Keeping one-side fixed to realise the marginal constraints, each node with a DOF can be considered to hold the sensors. A brief overview can be seen in Figure 5. The first three frequencies and modes can be seen in Table 1 and are also demonstrated in Figure 6. These were deduced considering the MOHGPSO load, and a quinary sensor set was planted in the inceptive manifestation. The attribute setup of the posited MOHGPSO was achieved by deploying GA as follows: the community length was set to approximately 49∼501 while constraining the maximal generation count to 99∼999. Maintaining a stochastically uniform selection procedure and keeping tolerance near about $10^{-6}∼10^{-3}$, the dispersed crossover method was adjusted to 0.9∼0.99. The Gaussian mutation assignment was kept around 0.009∼0.09. The delineated setup can be resolved through the forth mentioned entities: the structural DOF count, the sensor population considered, and most crucially, the intricacy of the OSP scenario.

**Figure 7:**
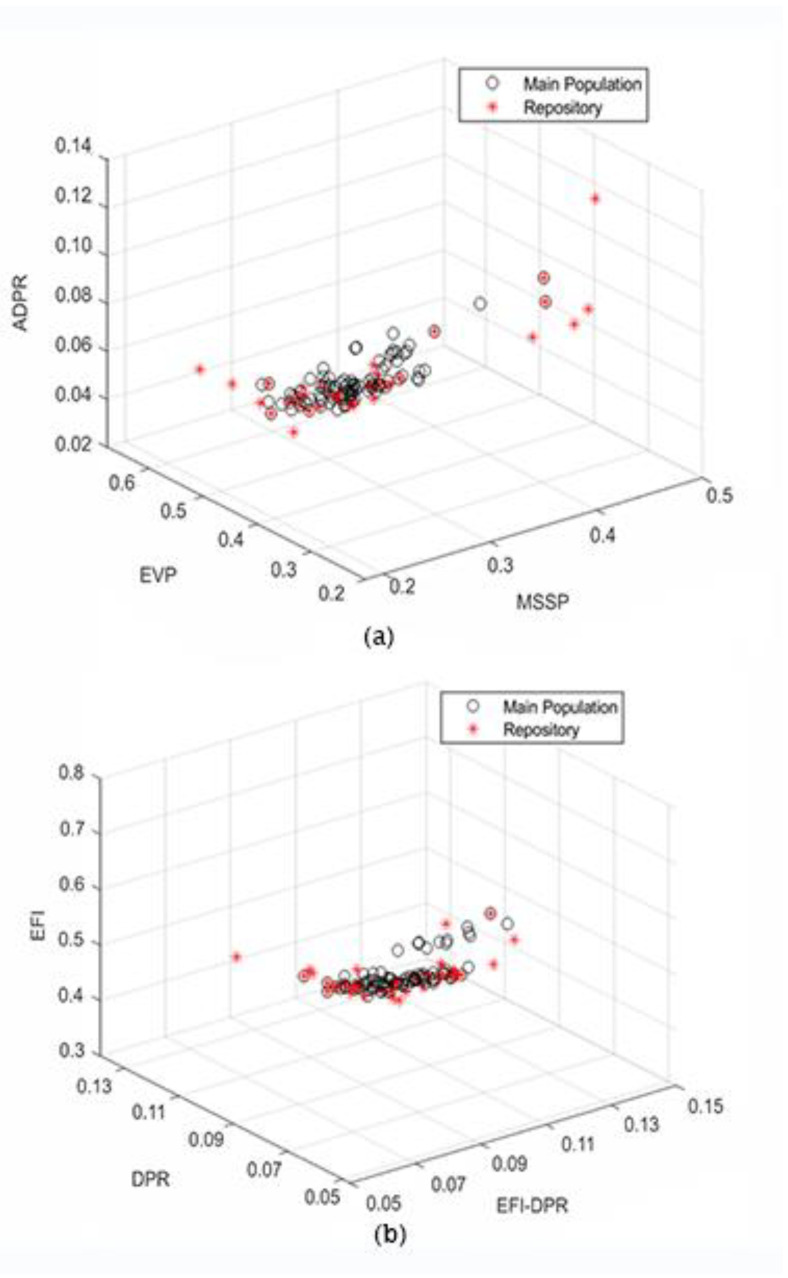
(**a**) Outcomes obtained through ADPR, EVP, and MSSP coplotted. (**b**) Outcomes obtained through EFI, DPR, and EFI-DPR coplotted.

In the discussed methodology, the iterative process is deployed to pick out the most optimal sensor placement orientation among the Pareto front solutions obtained from the analysis of the combined multi-objective problem through the proposed MOHGPSO, as presented in Figure 7a,b. Six sensor placement functions have been evaluated, focusing on the single point of significance in the first step of the initial case. Corresponding to the advancement in the iterative step, each function is assessed consecutively. However, the combined fitness function is dominated by the OSP methods of EfI, EVP, and MSSP, whereas the influence of the remanent methods on the MOHGPSO is considerably recessive.

Superiority has been visualised mostly through counterpoised performance and overall effectiveness. As a result, the sensor orientations determined by the proposed method were not always the best in all categories, but they may have been better than those acquired by other individual generic OSP methods. The proximal convergence of the fitness values for the groups of functions coplotted, as shown at the top of Figure 7a,b, reinforces the credibility of the proposed approach in determining the best fitness value. It is noteworthy that the iterative process required only seven steps, but that is greatly attributed to the fewer DOFs in the system considered. Thus, the upper hand of this proposed optimization algorithm is divulged in the form of reflexive determination of the most relevant sensor placement configurations in the combined fitness function without any artificial interruptions.

The forenamed five OSP criteria in Section 5.1 are employed in the evaluation of the optimality of the sensor orientations corresponding to their respective metrics. The resultant placements achieved through the aforementioned OSP functions in the posited MOHGPSO algorithm are registered in the form of Table 2. Apart from the DPR and its averaged counterpart, ADPR, the outcomes achieved through remanent OSP functions are contrasting. The final sensor orientations approved are the result of a synthetic combination of the output of the previously discussed six classical methods, i.e., the one that projects maximum concordance. They are further testified through the implementation of GRA imposed in the external archive, described in Section 3.2. Furthermore, for the sensor outputs acquired through the sextet of OSP functions, the 5th, 11th, and 20th DOFs are observed to occur more routinely in cases of the Effective Independence and Mode Shape Summation Plot functions, in proportion with the optimization metrics of all the OSP functions. These three positions have greater relevance to the optimum solution, which is further reflected in the effect of the combination. The EfI method has been distinguished by exhibiting the finest performance vis-à-vis the indices of the DET of FIM, along with MAC. Nevertheless, the results are entirely unalike the remaining sensor positions substantiated through the RSP. The outcomes of EfI-DPR, along with the MSSP approaches, are the closest to the others. Nonetheless, the remnant necessities were inadequate. In these criteria, the remaining classes of sensor orientation outcomes could obtain only superior or inferior.

The phenomenon of primary significance has been that the ADPR, EVP, and MSSP in collaborative fitness methods, as depicted in Figure 7a, emerge with most of the sensor counts. Consequently, it is perceived as an outcome of using the distinctive OSP functions. It partially accentuates the fact that the maximal contribution of the EfI, merged with DPR for the quinary set of sensors, has been the occurrence of the 5th, 6th, 12th, and 20th positions in the conclusive outcomes. This particular detection is accredited to the cumulative aftermath of the varying multiple objectives in combined fitness. The performance of the DET has been observed to be proportional to the number of sensors involved. A larger number of sensors demonstrating a denser configuration can induce irrelevant recurrence in the sensor configuration data. Comparison between the sensor locations achieved through the OSP functions and distinct sensor counts reveals that the Ratio of Similar Position pointer manifests a superior collective outcome, as per estimations, approximately 68% of the RSP can be seen as analogous to their respective sensor orientations obtained through MOHGPSO. This algorithm was augmented through the combination of classical algorithms, viz., the HGPSO, along with the GRA and some concepts of FDM, to artificially counterbalance and scrutinize all OSP conduction metrics, as demonstrated in Table 2. Its performance can be wholly attributed to the collective execution of the aforementioned OSP methodologies in addition to the component algorithms, collaboratively pivoted over the achievement of the sensor placement criteria, as discussed in Section 5.1. Relatively exemplary performance has been registered apropos of the MSE. Thus, from a general viewpoint, the posited MOHGPSO can be said to perform effectively.

Based on the data in Table 2 and Table 3, it appears that the proposed method in this paper may have limited advantages compared to the EFI method and the Novel Sensor Placement Algorithm method. The proposed method’s performance, as indicated by the evaluation criteria in Table 2, is similar to or slightly better than the EFI method in terms of DET, MAC, MSE, and SDI. However, it is important to note that the results achieved by the proposed method are inferior to those of the Novel Sensor Placement Algorithm method in terms of DET, SDI, and RSP.

The reason for this discrepancy in performance could be attributed to several factors. It is possible that the proposed method may not fully capture the complexities and nuances of the sensor placement problem, leading to suboptimal results compared to the Novel Sensor Placement Algorithm method. Additionally, the proposed method may have limitations in terms of its optimization approach, or the specific criteria used for evaluating sensor orientations.

Further analysis and explanation from this paper would be required to fully understand the reasons behind the observed performance differences and to provide a more comprehensive assessment of the proposed method’s advantages and disadvantages compared to other methods.

### 5.3. Fixed Wing

**Figure 8:**
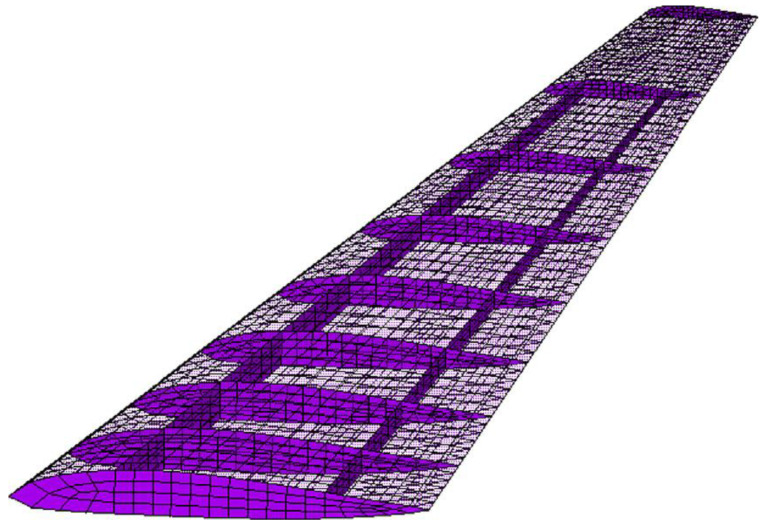
The fixed wing’s determinate entity model.

**Figure 9:**
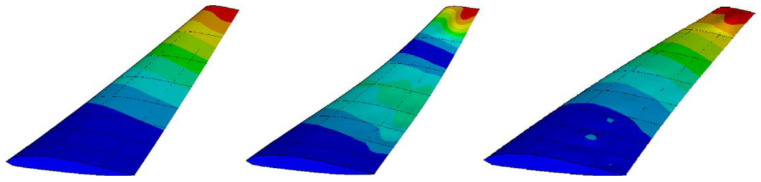
The fixed wing modal shapes.

An aeroplane is undoubtedly an intricate complex of numerous integrated systemic constituents, each meticulously designed to manoeuvre a predetermined section as its purpose. These structural subsystems are vulnerable to the risk facets involved in the flight undertakings, particularly the wings, which are subjected to severe circumstances encompassing myriad reverberations and vibratory jolts. For the examination of the robustness and structural coherence of the wings, a specified wing of precisely two and one-half meters in gauge, comprising ribs, skins, and spars, was selected as a further paradigm to evaluate the posited MOHGPSO. The marginally constrained FE model, i.e., with a riveted pinion at the core, has been exemplified in Figure 8. Expecting an expedient evaluation, specifically the out-of-plane DOF directions were considered as nominal sensor positions in Particle Swarm. Additionally, the modes and frequencies in Table 2, and also demonstrated in Figure 9, were used as the HGPSO load, while placing the ten considered sensors within the depicted wing framework. During MOHGPSO’s convergence at the 9th step, the contributions of the EfI and EVP are perceived to have an overall dominance.

The evaluation parameters in the fixed-wing aircraft experiment may be inconsistent with the spring–mass system due to the differences in the nature and complexity of the two systems. The fixed-wing aircraft is a complex system with numerous integrated components, such as wings, ribs, skins, and spars, designed to withstand various flight conditions and vibrations. The evaluation parameters for the fixed-wing aircraft experiment may focus on factors specific to aircraft structures, such as robustness, structural coherence, and the ability to withstand reverberations and vibratory jolts. On the other hand, the spring–mass system is a simplified model used to study the dynamics of a mass attached to a spring, which may have different evaluation parameters, such as natural frequency, damping ratio, and mode shapes. The inconsistency in evaluation parameters between the fixed-wing aircraft experiment and the spring–mass system may be due to the different objectives and requirements of the two systems.

The fitness function cumulatively approaches estimations of 0.79 with advancements in step. A sensor orientation computed through the conglomeration of all the OSP methods is seen to be collectively focused mostly on terminal sections of the wing. Regarding the quantification procedure of these methods, this finding ensures a considerable MSE influence.

### 5.4. Optimized Sensor Positions in Fixed Wing Aircraft Experiments

Optimized sensor positions in fixed-wing aircraft experiments can be determined using advanced optimization techniques and algorithms. One approach is to use the MOHGPSO algorithm, which considers multiple objectives and constraints to find the optimal sensor positions. OSP methods can also be employed to identify the most effective locations for sensors on the aircraft structure. These methods take into account factors such as the structure’s vulnerability to risk factors, the efficiency of data collection, and the conservation of sensor nodes’ energy. The optimized sensor positions can be determined by analyzing the results of the optimization algorithms and selecting the positions that provide the best performance in terms of structural health monitoring and the durability of the aircraft.

Sensor orientation determined by all OSP techniques is primarily focused on wing terminals. This indicates a significant MSE effect on these approaches’ quantification. The MOHGPSO has six visible sensor orientations at the wing’s front and back. Three neighboring sensors acquired using EfI functions have totally distributed locations, demonstrating the hypothesized combined processes’ effectiveness and the possibility of recurrence improvement. This supports the consideration of Table 3’s criteria. Even slight decreases in the DET of the FIM index of the proposed algorithm increase SDI performance by improving sensor orientations, proving its practicality.

Using data from sensors placed at the respective optimal configuration in the case of each OSP method, the respective modal properties are deduced and then treated as a vector of decision variables. During the course of the simulation, two systems, the spring–mass system and the fixed-wing of the airplane, are presented for optimal sensor placement by the proposed multi-objective methodology. It shows the frequency of the three vibrational modes calculated for the system’s spring–mass and fixed wing. These modes are calculated for 20 degrees of freedom. These values were deduced by considering a MOHGPSO load and a sensor set considered for inspection. After this, at the various sensor positions, the MOHGPSO dispenses six visibly dispersed sensor orientations placed at the frontal and rear extremities of the wing framework. Through the EfI functions, the three neighbouring sensors obtained have thoroughly scattered positions, projecting the efficacy of the posited combined functions while indicating the feasibility of recurrence improvement. This finding can account for the evaluation of the aforementioned criteria as listed in Table 3. Even trivial reductions in the DET of the FIM index of the proposed algorithm improved the performance of SDI by enhancing the sensor orientations, indicating the feasibility of the presented method.

## 6. Conclusions and Future Work

This paper addresses the challenges of a novel MOHGPSO algorithm for optimal sensor placement in SHM systems. It employs six established OSP methods to generate a Pareto front, which is systematically analyzed and archived through GRA and FDM. The proposed approach autonomously determines the most relevant sensor placements in the combined fitness function without artificial interventions, highlighting its superior performance. The study’s findings have implications for decision-makers in the engineering domain, providing comprehensive insights into the operation, design, and management of structures throughout their lifetimes. By achieving a Pareto front, the study enables decision-makers to evaluate and select sensor placements based on multiple criteria simultaneously, maximizing the effectiveness and efficiency of SHM systems. It also contributes to the preference of multi-objective optimization in SHM over the single-objective approach, as it allows for a trade-off of distinct objectives and avoids overlooking potential optimal sensor placement methods. The MOHGPSO algorithm’s convergence and coverage in modelling Pareto optimal solutions contribute to the efficient determination of the Pareto optimal solution set without the need for extra weights or aggregation. The method requires the assignment of weight factors to each aim, which can be arbitrary in the absence of supported computation or reference. Revising the weight factors for a combination of objectives incurs high computational costs. The proposed method aims to find a precisely explicit Pareto optimal solution set with minimal function evaluation. However, there is a trade-off between coverage and convergence, requiring an effective approach to counterbalance these factors. Results from assessments on a spring–mass system and fixed-wing subsystem highlight MOHGPSO’s superiority over generic methods, demonstrating reflexive determination of relevant sensor configurations. Future suggestions include synchronized recombination of OSP functions and leveraging Artificial Intelligence and Big Data analytics for optimal solutions, enhancing efficiency and accuracy. Multi-objective optimization algorithms, like MOHGPSO, are acknowledged for achieving a superior trade-off in addressing distinct objectives compared to single-objective methods.

## Figures and Tables

**Table 1:** Frequencies implemented in the demonstrated examples.

	1st	2nd	3rd
**Spring–mass system**	0.387 cycles/s (Hz)	1.14 cycles/s (Hz)	1.93 cycles/s (Hz)
**Fixed Wing**	24.3 cycles/s (Hz)	84.1 cycles/s (Hz)	141 cycles/s (Hz)

**Table 2:** Assessment of the sensor orientations achieved through the set of OSP functions considering the discussed criteria in the spring–mass setup.

	Sensor Positions	DET	MAC	MSE	SDI	RSP
Effective Independence	5, 6, 12, 13, 20	0.031	0.003	489.814	0.343	0.366
Driving Point Residue	16, 17, 18, 19, 20	0.000	0.786	77.774	0.255	0.568
Average DPR	16, 17, 18, 19, 20	0.000	0.786	77.774	0.255	0.568
EFI-DPR	12, 17, 18, 19, 20	0.000	0.571	254.940	0.481	0.599
Eigenvalue Vector Product	10, 11, 18, 19, 20	0.001	0.437	258.365	0.203	0.534
Mode Shape Summation Plot	5, 11, 18, 19, 20	0.021	0.292	445.806	0.637	0.601
Novel Sensor Placement Algorithm [23]	5, 6, 11, 12, 20	0.030	0.014	490.155	0.332	0.433
MOHGPSO (proposed)	12, 17, 4, 1, 6	0.031	0.013	491.009	0.331	0.431

**Table 3:** Assessment of the sensor orientations achieved through the set of OSP methods based on the discussed criteria in the fixed wing.

Features	$\mathrm{DET}\;(\times 10^{8})$	SDI	RSP
Effective Independence	1.301	0.684	0.432
Driving Point Residue	0	0.144	0.784
Average DPR	0	0.144	0.784
EFI-DPR	0	0.143	0.784
Eigenvalue Vector Product	0	0.144	0.784
Mode Shape Summation Plot	0	0.144	0.784
Novel Sensor Placement Algorithm	1.268	0.144	0.784
(Proposed) MOHGPSO	1.266	0.143	0.786

## Data Availability

All the data is available in this study.
